# Insecticidal Activity of Four Lignans Isolated from *Phryma leptostachya*

**DOI:** 10.3390/molecules24101976

**Published:** 2019-05-22

**Authors:** Yankai Li, Jiaqi Wei, Jiameng Fang, Wenbo Lv, Yufei Ji, Ahmed A.A. Aioub, Jiwen Zhang, Zhaonong Hu

**Affiliations:** 1 Institute of Pesticide Science, College of Plant Protection, Northwest A & F University, Yangling, Shaanxi 712100, China; liyankai1991@163.com (Y.L.); 18792539822@163.com (J.W.); jiameng_fang@163.com (J.F.); lvwenbo721@163.com (W.L.); jyf_1991@126.com (Y.J.); Ahmedaioub1991@gmail.com (A.A.A.A.); 2 Key Laboratory for Botanical Pesticide R & D of Shaanxi Province, Yangling, Shaanxi 712100, China; nwzjw@nwsuaf.edu.cn; 3 Plant Protection Department, Faculty of Agriculture, Zagazig University, Zagazig 44511, Egypt; 4 College of Chemistry & Pharmacy, Northwest A & F University, Yangling, Shaanxi 712100, China; 5 Key Laboratory of Integrated Pest Management on Crops in Northwestern Loess Plateau, Ministry of Agriculture, Yangling, Shaanxi 712100, China

**Keywords:** lignans, Phryma leptostachya, insecticidal activity, Mythimna separata, Plutella xylostella

## Abstract

A new lignan (**T4**) and three known lignans (**T1**, **T2**, and **T3**) were isolated from the methanol extract of the roots of *Phryma leptostachya* using bioassay-guided method, and their structures were identified as phrymarolin I (**T1**), II (**T2**), haedoxan A (**T3**), and methyl 4-((6a-acetoxy-4-(6-methoxybenzo[*d*][1,3]dioxol-5-yl)tetrahydro-1*H*,3*H*-furo[3,4–*c*]furan-1-yl)oxy)-1-hydroxy-2,2-dimethoxy-5-oxocyclopent-3-ene-1-carboxylate (**T4**) byNMR and ESI-MS spectral data. Bioassay results revealed that haedoxan A exhibited remarkably high insecticidal activity against *Mythimna separata* with a stomach toxicity LC_50_ value of 17.06 mg/L and a topical toxicity LC_50_ value of 1123.14 mg/L at 24 h, respectively. Phrymarolin I and compound **T4** also showed some stomach toxicity against *M. separata* with KD_50_ values of 3450.21 mg/L at 4 h and 2807.10 mg/L at 8 h, respectively. In addition, phrymarolin I and haedoxan A exhibited some stomach toxicity against *Plutella xylostella* with an LC_50_ value of 1432.05 and 857.28 mg/L at 48 h, respectively. In conclusion, this study demonstrated that lignans from *P. leptostachya* are promising as a novel class of insecticides or insecticide lead compounds for developing botanical pesticides.

## 1. Introduction

*Phryma leptostachya* is a perennial herb and is widely found in the Himalayas, temperate Asia, and northern East America [1,2,3]. The plant is a traditional Chinese medicine (TCM), which is commonly used to treat inflammatory diseases, such as allergic dermatitis, gout, and itch [4]. In folk pesticides, *P. leptostachya* has been traditionally used as a natural botanical insecticide in East Asia [5,6,7,8]. For instance, it was used to drive or kill mosquitos and flies in the southwest district of China [9]. Previous phytochemical investigations showed that this plant is rich in lignans, many of which have a unique oxygenated 3,7-dioxabicyclo[3.3.0]octane skeleton, such as phrymarolin I, II, and haedoxan A [1,10,11,12]. In general, these lignans are considered as the main insecticidal active ingredients in *P. leptostachya* [13,14].

These lignans from *P. leptostachya* exhibited highly efficient insecticidal activities against a variety of pests [6,15,16], which aroused our interest. For example, haedoxan A displayed remarkably high insecticidal activity against the housefly, which was approximate with common synthetic pyrethroids [17,18,19,20]. Phrymarolins I and II also showed considerable synergistic activities to pyrethrin and carbamate pesticides [3]. Natural products extracted from plants play an important role in crop protection, some of which have been developed as botanical pesticides, such as azadirachtin, matrine, and celangulin [21,22,23]. Accordingly, development of a new botanical pesticide with lignans from *P. leptostachya* as the main insecticidal ingredients has a broad prospect. Currently, only a few insecticidal active ingredients have been isolated and identified from *P. leptostachya*, and their insecticidal spectrum is limited. Therefore, this study aimed to isolate some insecticidal compounds from *P. leptostachya* using bioassay-guided method and expand their insecticidal spectrum. Ultimately, a novel lignan (**T4**) and three known lignans (phrymarolin I, II, and haedoxan A) were isolated from the roots of *P. leptostachya*, and their structures have been identified byNMR and ESI-MS. Also, this study demonstrated that the insecticidal activities of the four compounds against *Mythimna separata*, *Plutella xylostella*, *Tetranychina harti*, *Aphis citricola,* and *Trialeurodes vaporariorum.*

## 2. Results and Discussion

### 2.1. Structural Elucidation 

The chemical structures of compounds **T1, T2,** and **T3** from *P. leptostachya* were confirmed by ^1^H-NMR, ^13^C-NMR (Table S1) and ESI-MS (Figures S1–S3), and compound **T4** was confirmed by 1D-NMR (Figures S5–S7), 2D-NMR (Figures S8–S10) and HR-ESI-MS (Figure S4), and their structures were shown in Figure 1. 

Compound **T1** has been identified as phrymarolin I (molecular formula: C_24_H_24_O_11_) by comparison of its spectral data with published paper [6].

Compound **T2** has been identified as phrymarolin II (molecular formula: C_23_H_22_O_10_) by comparison of its spectral data with published paper [7].

Compound **T3** has been identified as haedoxan A (molecular formula: C_33_H_34_O_14_) by comparison of its spectral data with published paper [16].

**Figure 2:**
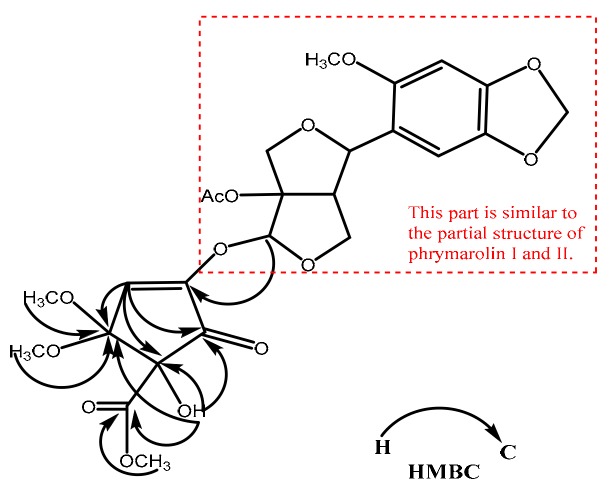
Key HMBC long-range correlations between C and H atoms of compound **T4**.

Compound **T4**, white solid, MP: 63.5–65.6 °C, [*α*] ^25^_D_: +115.39 (*c* = 0.10 g/100 mL, ethyl acetate). The UV max spectra of compound **T4** were 203, 234 and 299 nm, which were similar to that of phrymarolin I (**T1**), II (**T2**) and haedoxan A (**T3**) (Figure S11). The molecular formula was calculated as C_25_H_28_O_14_ by the analysis of its 1D-NMR, 2D-NMR and HR-ESI-MS (*m*/*z* 575.1361 [M + Na]^+^; calcd for C_25_H_28_O_14_Na, 575.1371) data. In the ^13^C, DEPT 135° and HSQC-NMR spectra, 5 CH_3_, 3 CH_2_, 6 CH, and 11 quaternary carbon atoms were observed. The compound showed the ^1^H-NMR and ^13^C-NMR signal (Table 1) of 1-(6-methoxybenzo[*d*][1,3]dioxol-5-yl)dihydro-1*H*,3*H*-furo[3,4-*c*]furan-3a(4H)-yl acetate (i.e., positions 6–8, 1′–10′, 7-OCOCH_3_ and 6′-OMe), which was identical to part structural signal of the known natural products phrymarolin I and II (Figure 2, Table S1) [6,7]. Moreover, the short-range correlation between C and H atoms in the HSQC spectrum clarified that δ_H_ 6.07 (s, 1H) correspond with δc 103.74 (C-6); δ_H_ 5.79 (s, 1H) correspond with δc 108.31 (C-2); δ_H_ 3.69 (s, 3H) correspond with δc 53.03 (4-COOC’H_3_); δ_H_ 3.39 (s, 1H) and 3.35 (s, 1H) correspond with δc 52.64 and 52.05 (3-OMe), respectively (Figure S9). The long-range correlation between C and H atoms in the HMBC spectrum clarified that δ_H_ 6.07 (s, 1H) correspond with δc 179.43 (C-1); δ_H_ 5.79 (s, 1H) correspond with δc 195.81 (C-5), 103.47 (C-3) and 86.57 (C-4); δ_H_ 5.00 (s, 1H) correspond with δc 195.81 (C-5), 171.31 (4-C’OOCH_3_), 103.47 (C-3) and 86.57 (C-4); δ_H_ 3.69 (s, 3H) correspond with δc 171.31 (4-C’OOCH_3_); δ_H_ 3.39 (s, 1H) and 3.35 (s, 1H) correspond with δc 103.47 (C-3), respectively (Figure 2 and Figure S10). Hence, the relative structure of compound **T4** was identified as methyl 4-((6a-acetoxy-4-(6-methoxybenzo[*d*][1,3]dioxol-5-yl)tetrahydro-1*H*,3*H*-furo[3,4-*c*]furan-1-yl)oxy)-1-hydroxy-2,2-dimethoxy-5-oxocyclopent-3-ene-1-carboxylate.

### 2.2. Insecticidal Activity

The bioassay results showed that haedoxan A (**T3**) exhibited the most excellent insecticidal activity against *M. separata* with 100% stomach and topical toxicity at any test time. Phrymarolin I (**T1**) and compound **T4** also exhibited some stomach toxicity against *M. separata* with a knockdown rate of 66.7% at 4 h and 95.8% at 8 h, respectively. Whereas the stomach toxicity of compound **T4** was only 25.0%, and phrymarolin I (**T1**) was even completely lost at 24 h (Table 2). Furthermore, the insecticidal activity of the three compounds against *M. separata* was further determined, and the results are given in Table 3. As shown in Table 3, haedoxan A (**T3**) exhibited remarkably high insecticidal activity against *M. separata* with a stomach toxicity LC_50_ value of 17.06 mg/L and a topical toxicity LC_50_ value of 1123.14 mg/L at 24 h, respectively. Importantly, the stomach toxicity LC_50_ value of haedoxan A (**T3**) was comparable with the commercial pesticide indoxacarb (20.73 mg/L). Phrymarolin I (**T1**) and compound **T4** also showed some stomach toxicity against *M. separata* with KD_50_ values of 3450.21 mg/L at 4 h and 2807.10 mg/L at 8 h, respectively. 

We also determined the insecticidal activity of these compounds against *P. xylostella*, and the results are shown in Table 4 and Table 5. As shown in Table 4, phrymarolin I (**T1**) and haedoxan A (**T3**) exhibited some insecticidal activity against *P. xylostella* with a stomach toxicity of 50.0% and 60.0% at 48 h, respectively. Subsequently, the insecticidal activity of the two compounds against *P. xylostella* was further determined. Data obtained from Table 5 showed that phrymarolin I (**T1**) and haedoxan A (**T3**) exhibited some stomach toxicity against *P. xylostella* with an LC_50_ value of 1432.05 and 857.28 mg/L at 48 h, respectively. However, none of these compounds exhibited topical toxicity against *P. xylostella* at the concentration of 1.0 mg/mL. To our knowledge, this is the first report on the insecticidal activity of lignans from *P. leptostachya* against *P. xylostella*. Unexpectedly, phrymarolin II (**T2**) showed no insecticidal activities against *M. separata* and *P. xylostella.*

To expand the insecticidal spectrum of these compounds from *P. leptostachya,* we further determined their insecticidal activities against *T. harti*, *A. citricola*, and *T. vaporariorum* using the slide-dip method [24], dip method [25], and leaf-dipping method [26], respectively. Unfortunately, these compounds did not show any toxic activities against the three test insects at the concentration of 1.0 mg/mL. These results indicated that lignans from *P. leptostachya* may not be effective against piercing-sucking mouthparts insects, but they showed good control effects on lepidopteran insects.

The trimer haedoxan A (**T3**) showed the most excellent insecticidal activities against *M. separata* and *P. xylostella*. Nevertheless, the insecticidal activities of the dimers phrymarolin I (**T1**) and II (**T2**) were weak. The result was similar to previous reports [27,28,29]. Similar insecticidal activities were also observed with stilbenes, a class of similar molecules to lignans, on *Spodoptera littoralis* and *Leptinotarsa decemlineata*. Among them, the tetramers vitisin A and B displayed the most excellent insecticidal activities compared with other stilbenes [30,31]. In summary, these results implied that the number of polymers of compounds may have a significant impact on their insecticidal activity, and they may exhibit the highest insecticidal activity in the form of trimers or tetramers.

## 3. Materials and Methods

### 3.1. Instruments

HPLC was performed using an Elite P230 system (Dalian Elite Analytical Instrument Co., Ltd., Dalian, China). ESI-MS was measured on an LTQ XL linear ion trap mass spectrometer (Thermo, Wilmington, MA, USA). HR-ESI-MS was obtained using an AB SCIEX Triple TOF 5600+ spectrometer (AB SCIEX, Boston, MA, USA). Melting point was measured on a WRR melting point apparatus (Shanghai Jingke Instrument Co., Ltd., Shanghai, China) and was uncorrected. Optical rotation was performed using an Anton Paar MCP 300 polarimeter (Anton Paar Opto Tec GmbH, Seelze, Germany) and was uncorrected. 1D (^1^H, ^13^C and DEPT 135°) and 2D (^1^H-^1^H COSY, ^1^H-^13^C HSQC and ^1^H-^13^C HMBC)-NMR spectra were performed using a Bruker Avance III 500 MHz instrument (Bruker, Billerica, MA) with CDCl_3_ (**T1**, **T2**, and **T3**) or C_3_D_6_O (**T4**) as the solvent and TMS as the internal standard.

### 3.2. Plant Materials

In September 2016, the roots of *P. leptostachya* were collected from Liaoyuan City, Jilin Province, China. The plant material was identified by Dr. Hua Yi and then air-dried. A voucher specimen (No. NWAU2009-PL15) was deposited in College of Life Sciences, Northwest A & F University.

### 3.3. Extraction and Purification

The air-dried roots of *P. leptostachya* (9.5 kg) were powdered and then extracted with methanol (30 L × 3) under soak for 24 h. After being filtered and concentrated, the final weight of the methanol extract was 671 g. Subsequently, the extract was suspended in water (3 L) and extracted with ethyl acetate (3 L × 3) to afford ethyl acetate phase (220 g). The ethyl acetate phase was subjected to silica gel column chromatography (SGCCT) and eluted gradiently with petroleum ether-ethyl acetate (10:0, 9:1, 8:2, 7:3, 6:4, 5:5, 0:10, *v*/*v*) to obtain eleven fractions: A1-A11. Fraction A7 (30 g) was then subjected to SGCCT and eluted by the method described above to yield nine fractions: B1-B9. Fraction B6 (6.2 g) was further subjected to SGCCT and eluted gradiently with petroleum ether- acetone (10:0, 6:1, 3:1, 2:1, 1:1, 0:10, *v*/*v*) to yield eight fractions: C1-C8. Fraction C2 and C5 were subjected to a semi-preparative HPLC with a mobile phase of methanol-water (75:25, *v*/*v*) to obtain compounds **T1** (185 mg), **T2** (8 mg), and **T3** (115 mg). Fraction C7 was also subjected to the semi-preparative HPLC with a mobile phase of methanol-water (60:40, *v*/*v*) to yield compound **T4** (40 mg). The flow rate was 3.0 mL/min, and A_230_ nm was used for monitoring and collecting.

### 3.4. Bioassay of Insecticidal Activity

*M. separata* and *P. xylostella* were reared continuously with fresh wheat or cabbage leaves in our laboratory without insecticides, respectively. This feeding environment was controlled at temperature 25 ± 2 °C, relative humidity 75 ± 5% and photoperiod 12 L: 12 D. Compounds were dissolved separately in acetone to obtain required concentration. The stomach toxicity of these compounds was evaluated against the third instar larvae of *M. separata* and *P. xylostella* starved for 12 h using the leaf disc method [32]. Briefly, fresh leaf discs (0.5 cm × 0.5 cm) were treated separately with 1.0 μL solution of the compounds to get the toxic leaf discs. Subsequently, these toxic leaf discs were fed separately to the test insects. The topical toxicity was evaluated against the third instar larvae of *M. separata* and *P. xylostella* using the topical application method [33]. Briefly, the pronotum of test insects was treated separately with 1.0 μL solution of the compounds, and they were then reared with fresh leaves. Acetone and indoxacarb (a commercial insecticide) were used as negative and positive control, respectively. For each concentration, 24 insects with triplicate (24 × 3) were tested. The knockdown rate of test compounds against *M. separata* was recorded at 4 h and 8 h, and the corrected mortality was recorded at 24 h. Also, the corrected mortality of test compounds against *P. xylostella* was recorded at 24 h and 48 h, respectively. This experiment was carried out twice and the insecticidal activities of these compounds were displayed by KD_50_ or LC_50_ value, as well as 95% confidence interval.

## 4. Conclusions

In summary, we isolated a new lignan (**T4**) and three known lignans (phrymarolin I, II, and haedoxan A) from the roots of *P. leptostachya*, and we demonstrated that haedoxan A exhibited remarkably high insecticidal activity against *M. separata* with a stomach toxicity LC_50_ value of 17.06 mg/L and a topical toxicity LC_50_ value of 1123.14 mg/L at 24 h, respectively. Importantly, the stomach toxicity LC_50_ value of haedoxan A against *M. separata* was comparable with the commercial chemical pesticide indoxacarb (20.73 mg/L). Moreover, phrymarolin I and haedoxan A also exhibited some stomach toxicity against *P. xylostella* with an LC_50_ value of 1432.05 and 857.28 mg/L at 48 h, respectively. To our knowledge, this work is the first report on the insecticidal activity of lignans from *P. leptostachya* against *P. xylostella*. These results suggested that developing a novel class of insecticides or insecticide lead compounds based on lignans from *P. leptostachya* as the major insecticidal active ingredients is promising.

## Figures and Tables

**Figure 1:**
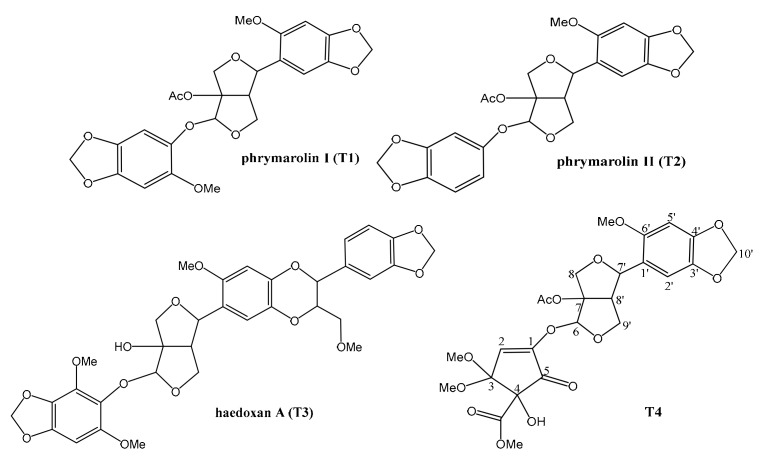
Lignans isolated from *P. leptostachya.*

**Table 1:** ^1^H-NMR (500 MHz) and ^13^C-NMR (126 MHz) data of compound **T4** measured in C_3_D_6_O.

Position	*δ_C_* (ppm)	*δ_H_* (ppm, *J* in Hz)
1	179.43	
2	108.31	5.79 (s, 1H)
3	103.47	
4	86.57	
5	195.81	
6	103.74	6.07 (s, 1H)
7	97.05	
8	76.01	3.85 (d, *J* = 11.2 Hz, 1H); 4.55 (d, *J* = 11.1 Hz, 1H)
1′	122.45	
2′	106.49	6.97 (s, 1H)
3′	142.23	
4′	148.60	
5′	95.45	6.71 (s, 1H)
6′	152.43	
7′	84.04	4.84 (d, *J* = 6.7 Hz, 1H)
8′	56.82	2.83 (s, 1H)
9′	71.33	4.16–4.29 (m, 2H)
10′	102.30	5.95 (s, 2H)
3-OMe	52.64	3.39 (s, 3H)
	52.05	3.35 (s, 3H)
4-OH		5.00 (s, 1H)
4-C′OOCH_3_	171.31	
4-COOC′H_3_	53.03	3.69 (s, 3H)
7-OC′OCH_3_	170.07	
7-OCOC’H_3_	20.89	2.05 (s, 3H)
6′-OMe	57.04	3.83 (s, 3H)

**Table 2:** Toxicity of compounds against *M. Separata.*

Compounds ^a^	Mode of Action	4 h Knockdown Rate (%)	8 h Knockdown Rate (%)	24 h Corrected Mortality (%)
**T1**	ST ^b^	66.7	0.0	0.0
	TT ^c^	8.3	0.0	0.0
**T2**	ST	0.0	0.0	0.0
	TT	0.0	0.0	0.0
**T3**	ST	100.0	100.0	100.0
	TT	100.0	100.0	100.0
**T4**	ST	0.0	95.8	25.0
	TT	0.0	0.0	0.0

^a^ The concentration of compounds was 10.0 mg/mL. ^b^ ST, stomach toxicity. ^c^ TT, topical toxicity.

**Table 3:** Insecticidal activity of compounds against *M. Separata.*

Compounds	Time (h)	Mode of Action	Toxicity Regession Equation (y = a + bx)	r	KD_50_/LC_50_^a^ (95% Confidence Interval) mg/L
**T1**	4	ST ^b^	y = −1.6517 + 1.8801x	0.9885	3450.21 (2568.05–4635.56)
		TT ^c^	-	-	>10,000
**T3**	24	ST	y = 2.9026 + 1.7027x	0.9814	17.06 (12.30–23.65)
		TT	y = −2.8244 + 2.5651x	0.9775	1123.14 (885.25–1425.01)
**T4**	8	ST	y = −2.7981 + 2.2615x	0.9819	2807.10 (2180.02–3614.31)
		TT	-	-	>10,000
**Indoxacarb**	24	ST	y = 1.9652 + 2.3051x	0.9896	20.73 (16.83–25.52)
		TT	y = 4.5199 + 2.2861x	0.9902	1.62 (1.15–2.29)

^a^ KD_50_ or LC_50_ mean the compound concentration when knocking down (4 and 8 h) or killing (24 h) insects to 50%, respectively. ^b^ ST, stomach toxicity. ^c^ TT, topical toxicity.

**Table 4:** Toxicity of compounds against *P. xylostella.*

Compounds ^a^	Mode of Action	24 h Corrected Mortality (%)	48 h Corrected Mortality (%)
**T1**	ST ^b^	0.0	50.0
	TT ^c^	0.0	0.0
**T2**	ST	0.0	0.0
	TT	0.0	0.0
**T3**	ST	10.0	60.0
	TT	0.0	0.0
**T4**	ST	0.0	0.0
	TT	0.0	0.0

^a^ The concentration of compounds was 1.0 mg/mL. ^b^ ST, stomach toxicity. ^c^ TT, topical toxicity.

**Table 5:** Insecticidal activity of compounds against *P. xylostella.*

Compounds	Time (h)	Mode of Action	Toxicity Regession Equation (y = a + bx)	r	LC_50_^a^ (95% Confidence Interval) mg/L
**T1**	48	ST ^b^	y = −0.2142 + 1.6521x	0.9813	1432.05 (1051.58–1952.23)
**T3**	48	ST	y = −0.9181 + 2.0178x	0.9772	857.28 (663.69–1108.28)
Indoxacarb	48	ST	y = 2.9102 + 2.3246x	0.9836	7.92 (6.25–10.05)

^a^ LC_50_ means the compound concentration when killing insects to 50%. ^b^ ST, stomach toxicity.

## Supplementary Materials

The following are available online.
