# Supplementary material for: Insecticidal Activity of Four Lignans Isolated from Phryma leptostachya
Source: Molecules. 2019 May 22;24(10):1976. doi: 10.3390/molecules24101976 (PMC6572576; doi:10.3390/molecules24101976)
Supplement: Supplementary file 1 [file molecules-24-01976-s001.pdf]

Supplementary Files

Insecticidal Activity of Four Lignans Isolated from *Phryma leptostachya*

Yankai Li [1,2], Jiaqi Wei [1,2], Jiameng Fang [1,2], Wenbo Lv [1,2], Yufei Ji [1,2], Ahmed A.A. Aioub [1,2,5], Jiwen Zhang [2,3] and Zhaonong Hu[1,2,4*]

1 Institute of Pesticide Science, College of Plant Protection, Northwest A & F University, Yangling, Shaanxi, 712100, P. R. China

2 Key Laboratory for Botanical Pesticide R & D of Shaanxi Province, Yangling, Shaanxi, 712100, P. R. China

3 College of Chemistry & Pharmacy, Northwest A & F University, Yangling, Shaanxi, 712100, P. R. China

4 Key Laboratory of Integrated Pest Management on Crops in Northwestern Loess Plateau, Ministry of Agriculture, Yangling, Shaanxi, 712100, P. R. China

5 Plant Protection Department, Faculty of Agriculture, Zagazig University, 44511 Zagazig, Egypt

* Authors to whom correspondence should be addressed; E-Mails: huzhaonong@nwsuaf.edu.cn; Tel./Fax: +86 29 87093987.

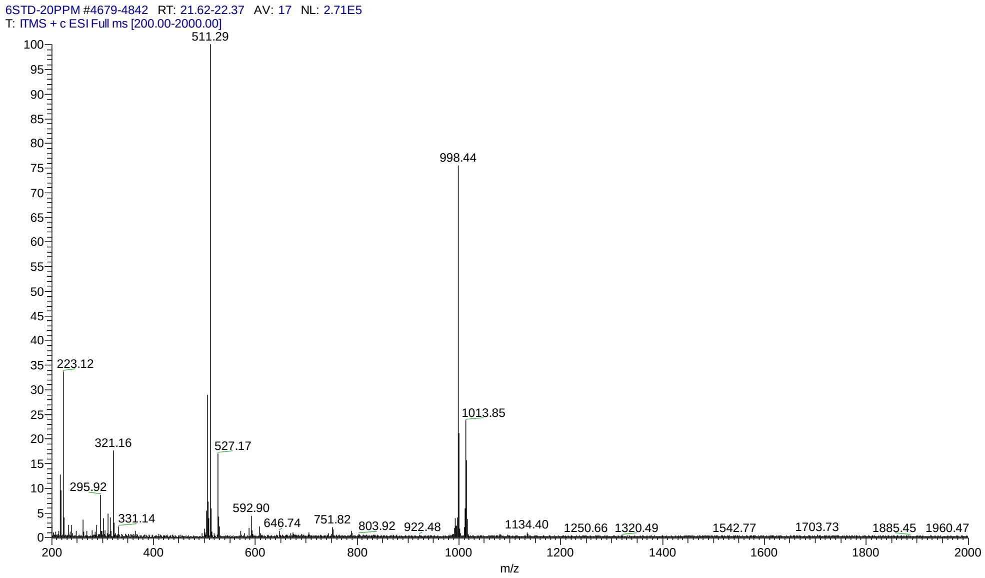

Figure S1. ESI-MS spectrum of compound **T1**.

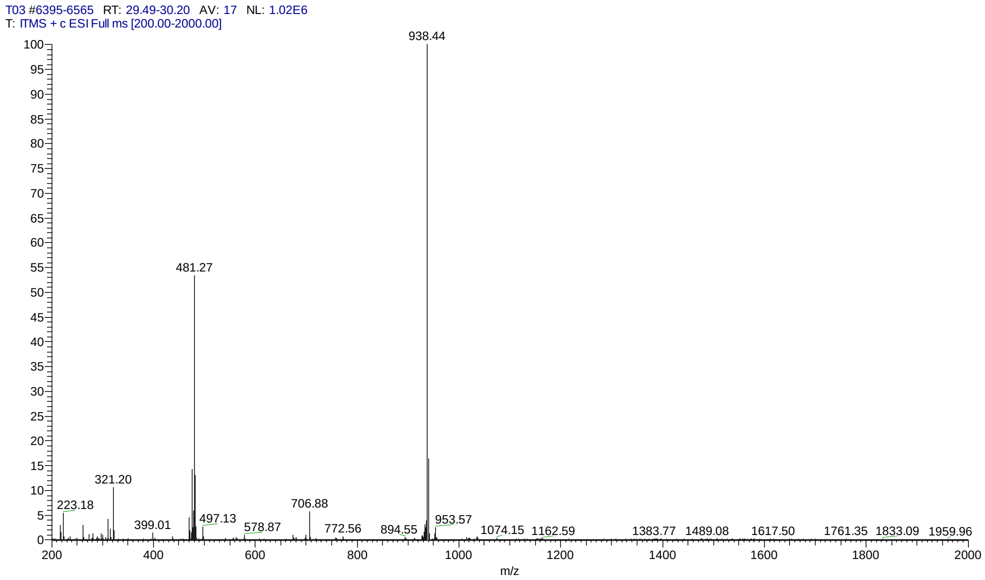

Figure S2. ESI-MS spectrum of compound **T2**.

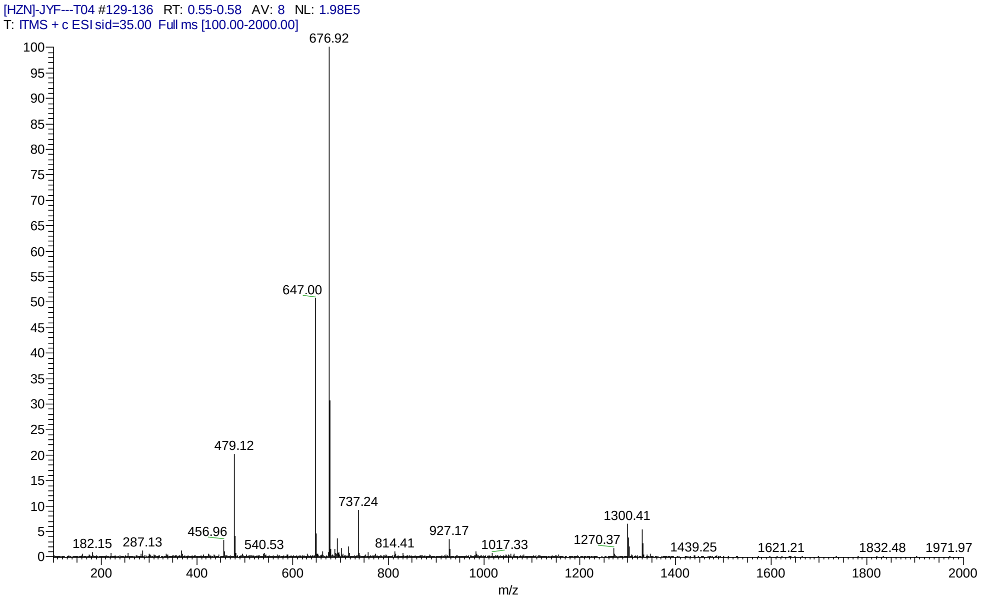

Figure S3. ESI-MS spectrum of compound **T3**.

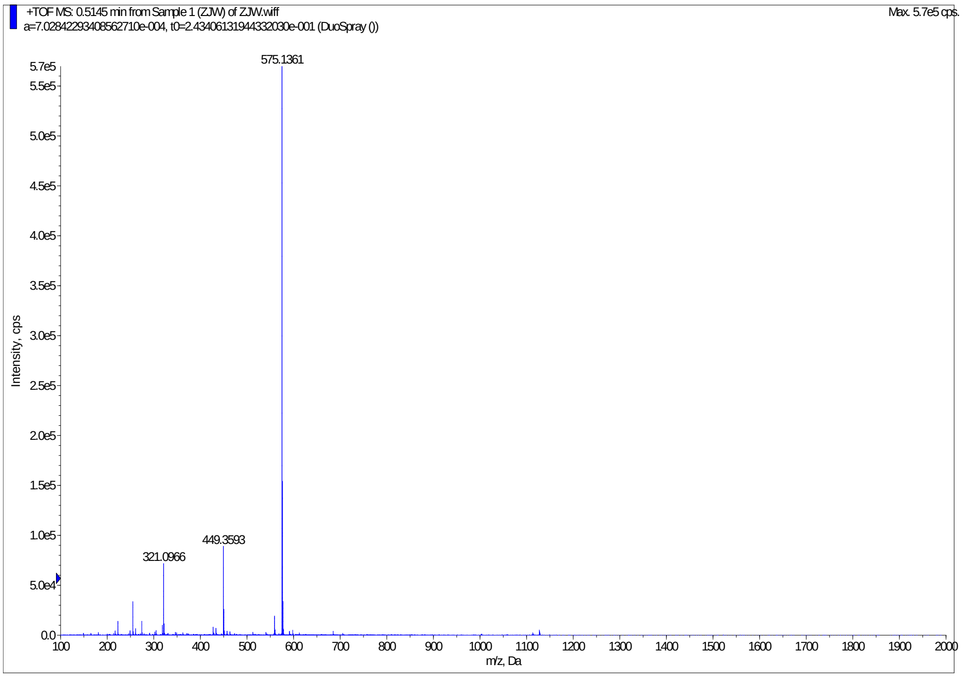

Figure S4. HR-ESI-MS spectrum of compound **T4**.

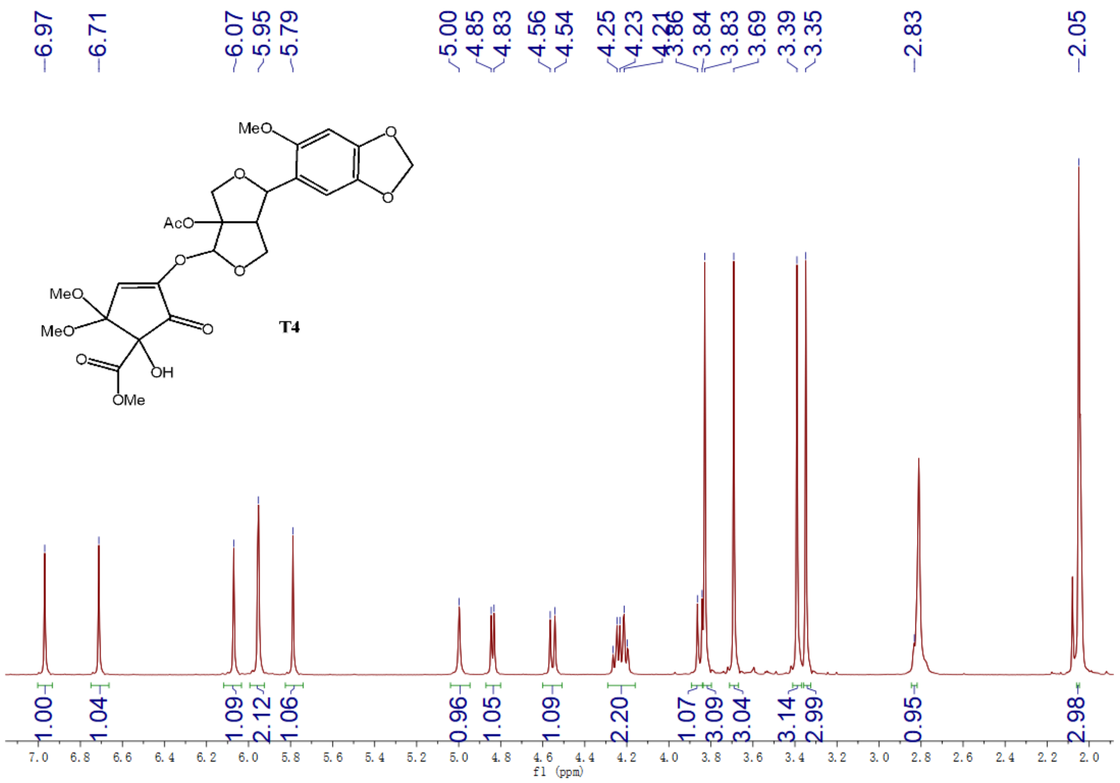

Figure S5. ^{1}H NMR spectrum of compound **T4** in C_3D_6O.

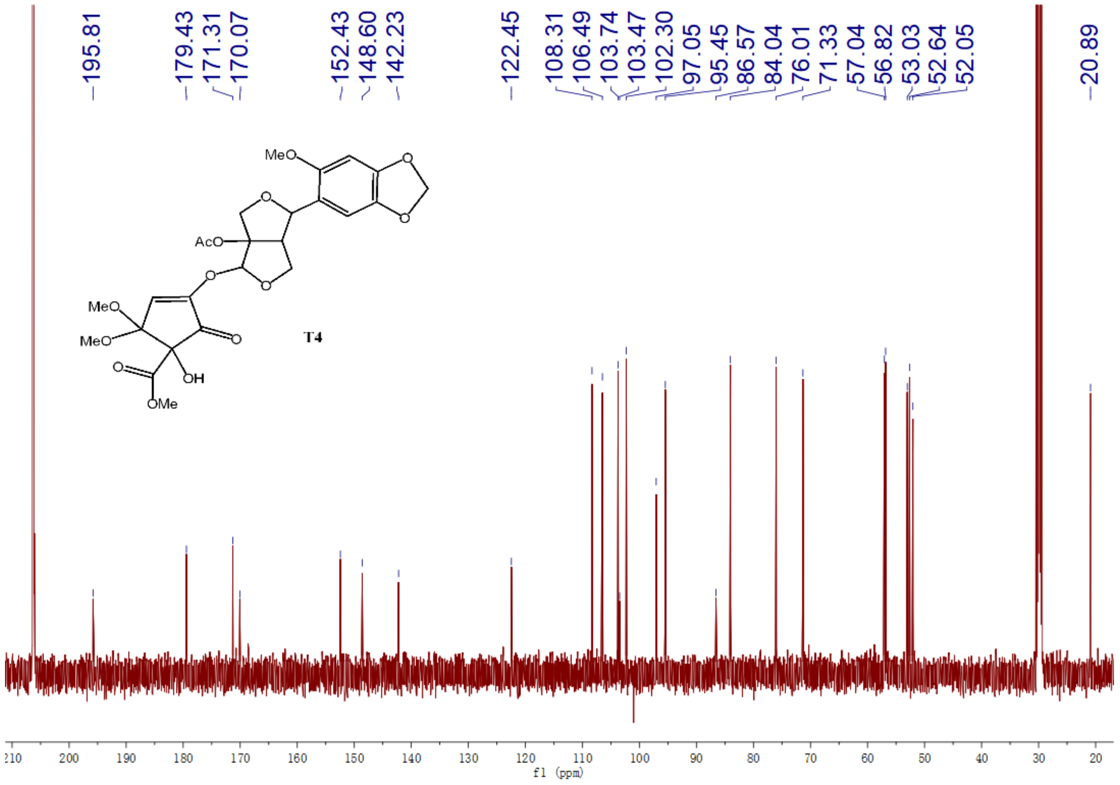

Figure S6. ^{13}C NMR spectrum of compound **T4** in C_3D_6O.

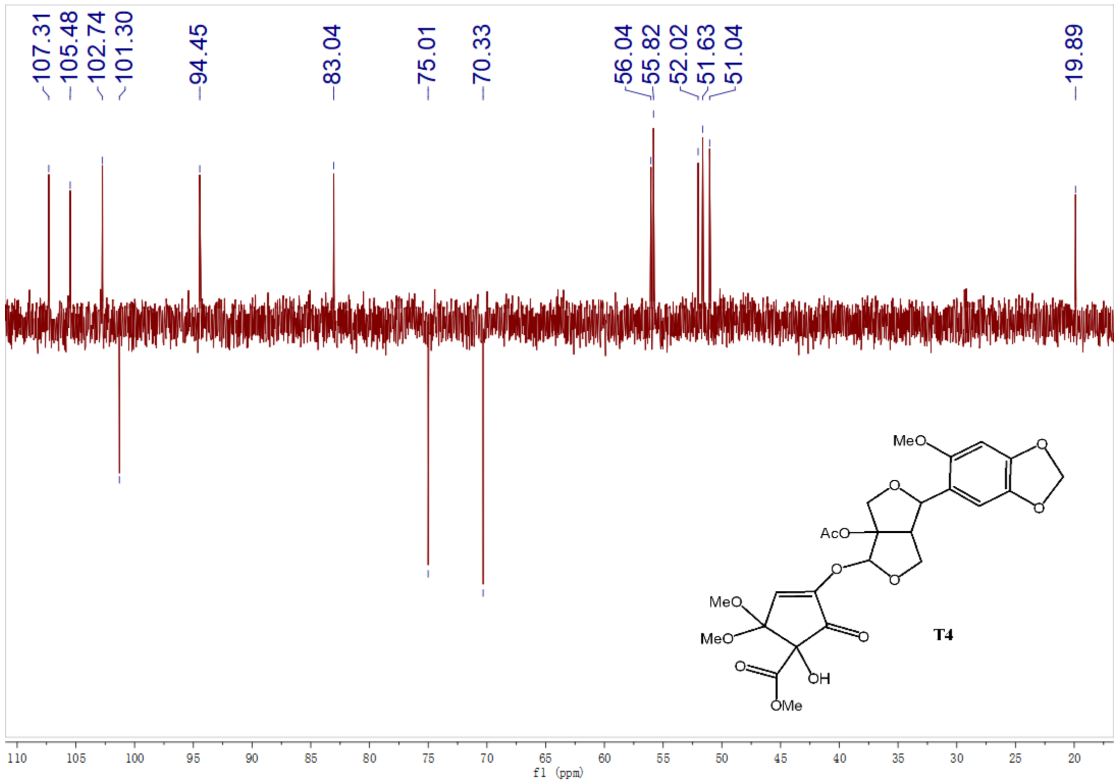

Figure S7. DEPT 135° spectrum of compound **T4** in C_3D_6O.

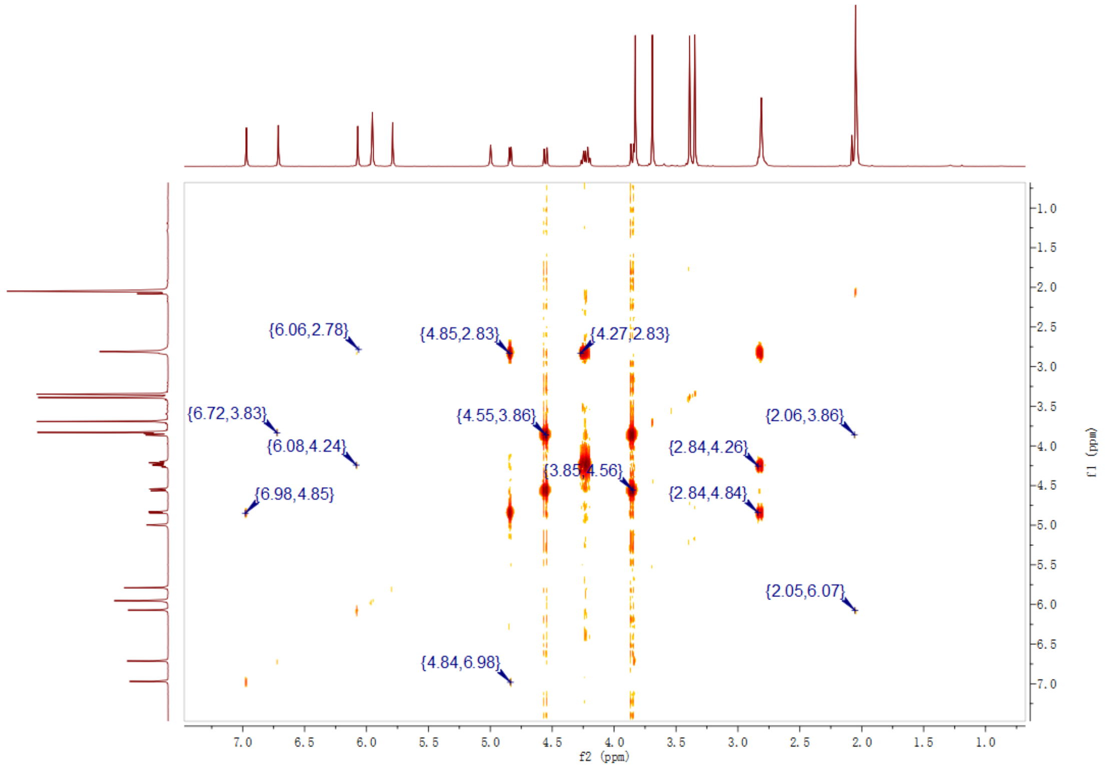

Figure S8. ^{1}H-^{1}H COSY spectrum of compound **T4** in C_3D_6O.

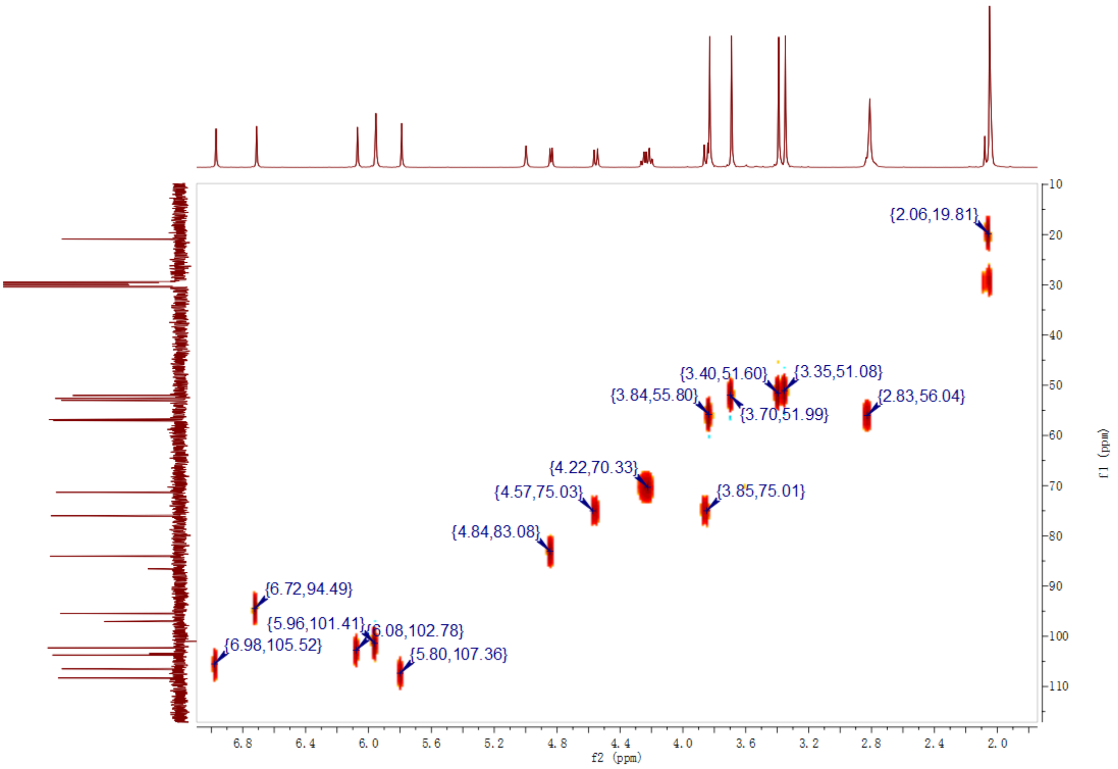

Figure S9. HSQC spectrum of compound **T4** in C_3D_6O.

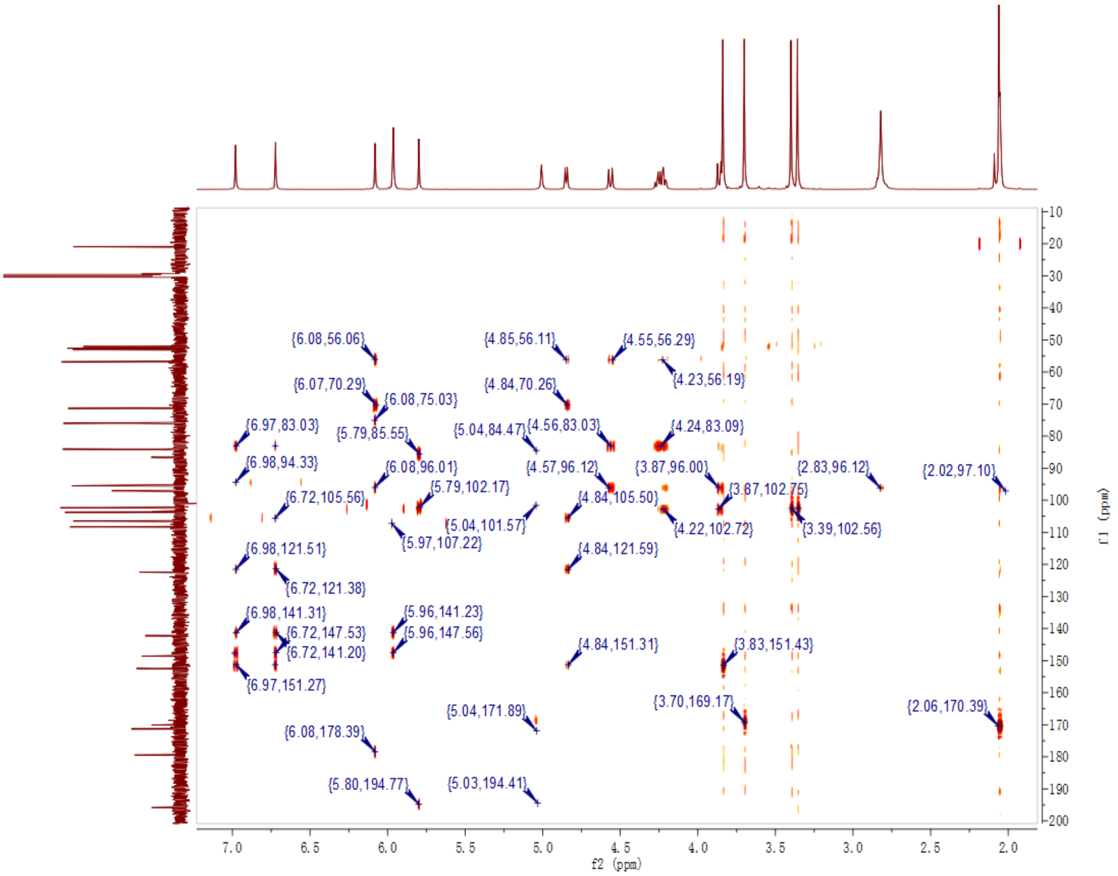

Figure S10. HMBC spectrum of compound **T4** in C_3D_6O.

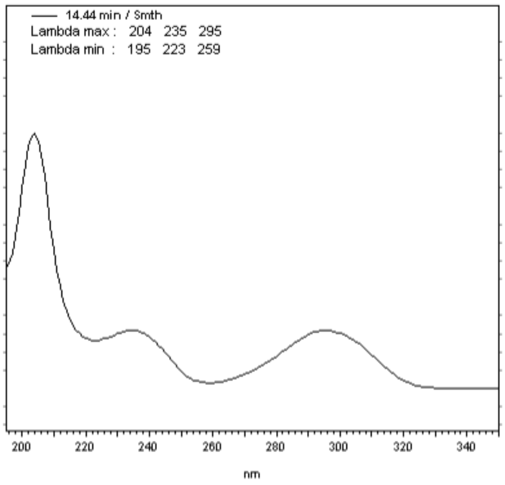

Phrymarolin I **(T1)**

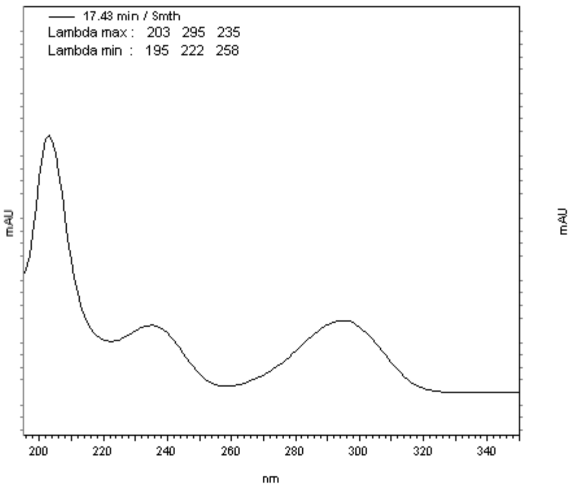

Phrymarolin II **(T2)**

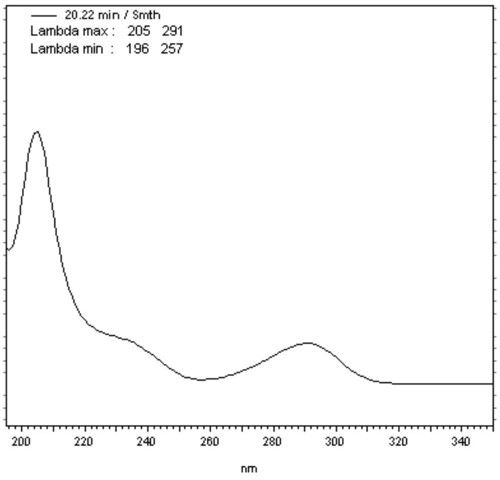

Haedoxan A (**T3**)

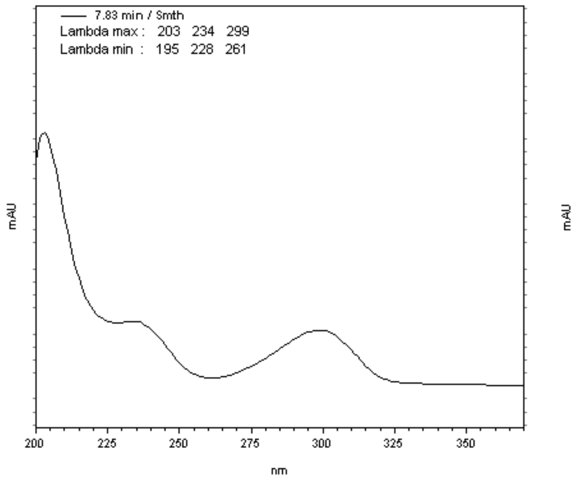

T4

Figure S11. UV spectra of compound **T1-T4**.

Table S1 ^{1}H NMR (500 MHz) and ^{13}C NMR (126 MHz) data of compounds **T1**, **T2** and **T3** measured in $CDCl_3$ (*J* in Hz).

Position	T1		T2		T3	
	δ_H (ppm)	δ_C (ppm)	δ_H (ppm)	δ_C (ppm)	δ_H (ppm)	δ_C (ppm)
1		147.59		152.01		131.31
2	6.52 (s, 1H)	106.45	6.58 (d, *J* = 2.3 Hz, 1H)	106.45		138.19
3		141.48		148.20		131.19
4		140.94		141.55		147.81
5	6.83 (s, 1H)	94.37	6.69 (d, *J* = 8.4 Hz, 1H)	108.16	6.29 (s, 1H)	89.78
6		143.02	6.49 (dd, *J* = 8.4, 2.3 Hz, 1H)	110.62		144.95
7	5.68 (s, 1H)	101.82	5.70 (s, 1H)	101.44	5.24 (s, 1H)	106.56
8		96.55		96.59		92.55
9	3.81 (d, *J* = 11.1 Hz, 1H); 4.61 (d, *J* = 11.1 Hz, 1H)	75.76	3.81 (d, *J* = 11.1 Hz, 1H); 4.60 (d, *J* = 11.1 Hz, 1H)	75.79	3.74 (d, *J* = 10.0 Hz, 1H); 4.30 (d, *J* = 9.6 Hz, 1H)	76.65
10	5.93 (s, 2H)	101.36	5.93 (s, 2H)	101.39	5.87 (d, *J* = 2.6 Hz, 2H)	101.40
1’		121.54		121.52		123.31
2’	7.05 (s, 1H)	104.03	7.05 (s, 1H)	103.19	7.31 (s, 1H)	107.77
3’		140.44		143.50		137.10
4’		145.75		147.66		143.05
5’	6.52 (s, 1H)	96.24	6.52 (s, 1H)	94.42	6.49 (s, 1H)	100.02
6’		151.56		151.58		150.83
7’	4.88 (d, *J* = 6.9 Hz, 1H)	83.10	4.87 (d, *J* = 7.0 Hz, 1H)	83.05	4.99 (d, *J* = 8.0 Hz, 1H)	83.98
8’	2.90 (t, *J* = 6.4 Hz, 1H)	57.11	2.84 (t, *J* = 6.6 Hz, 1H)	56.65	2.64 (t, *J* = 7.5 Hz, 1H)	57.98
9’	4.05 (dd, *J* = 9.1, 1.8 Hz, 1H); 4.40 (dd, *J* = 9.1, 7.0 Hz, 1H)	68.97	4.06 (dd, *J* = 9.2, 1.5 Hz, 1H); 4.29 (dd, *J* = 9.1, 6.9 Hz, 1H)	68.76	4.05 (dd, *J* = 9.1, 2.3 Hz, 1H); 4.59-4.54 (m, 1H)	71.92
10’	5.87 (dd, *J* = 4.4, 1.3 Hz, 2H)	101.32	5.92 (s, 2H)	101.36		
1’’						130.66
2’’					6.83-6.92 (m, 1H)	108.58
3’’						148.18
4’’						148.11
5’’					6.83-6.92 (m, 1H)	115.40
6’’					6.83-6.92 (m, 1H)	121.44
7’’					4.93 (d, *J* = 6.0 Hz, 1H)	77.35
8’’					4.00 (d, *J* = 5.3 Hz, 1H)	77.74
9’’					3.30 (dd, *J* = 11.0, 3.7 Hz, 1H); 3.58 (dd, *J* = 11.0, 2.2 Hz, 1H)	71.37
10’’					5.99 (s, 2H)	101.22
2-OCH_3					4.02 (s, 3H)	60.25
6-OCH_3	3.77 (s, 3H)	56.36			3.73 (s, 3H)	57.05
6’-OCH_3	3.74 (s, 3H)	56.35	3.77 (s, 3H)	56.38	3.78 (s, 3H)	55.89
$CH_3C’O$-		170.95		170.90		
$C’H_3CO$-	2.13 (s, 3H)	21.22	2.13 (s, 3H)	21.23		
9’’-OCH_3					3.35 (s, 3H)	59.68
